# Efficacy of rasagiline and selegiline in Parkinson’s disease: a head-to-head 3-year retrospective case–control study

**DOI:** 10.1007/s00415-017-8523-y

**Published:** 2017-05-26

**Authors:** Emanuele Cereda, Roberto Cilia, Margherita Canesi, Silvana Tesei, Claudio Bruno Mariani, Anna Lena Zecchinelli, Gianni Pezzoli

**Affiliations:** 1 Nutrition and Dietetics Service, Fondazione IRCCS Policlinico San Matteo, Viale Golgi 19, 27100 Pavia, Italy; 2 Parkinson Institute, ASST G. Pini-CTO, ex-ICP, Milan, Italy

**Keywords:** Parkinson’s disease, Levodopa, Selegiline, Rasagiline, Monoamine oxidase inhibitors

## Abstract

Monoamine oxidase type B (MAO-B) inhibitors, such as selegiline and rasagiline, can be used as monotherapy or adjuvant therapy to levodopa in Parkinson’s disease (PD). Data on long-term efficacy of MAO-B inhibitors are limited with no head-to-head comparison available to date. The aim of this case–control retrospective study was to analyze data from patients with PD attending the Parkinson Institute (Milan, Italy) over a 6-year period (2009–2015) and compare the effects of selegiline and rasagiline on levodopa treatment outcomes. Patients with PD treated with either selegiline (*n* = 85) or rasagiline (*n* = 85) for 3 years as well as a control group of patients (*N* = 170) who have never received MAO-B inhibitors, were matched for gender, disease duration (±1 year) and age (±1 year) at baseline assessment (ratio 1:1:2). The Unified PD Rating Scale and the Hoehn–Yahr staging system were used for clinical comparisons. At baseline, mean PD duration was 6.5 years and clinical features were comparable across all three groups. After a mean follow-up of approximately 37 months, no differences in clinical progression of motor and non-motor symptoms were observed between the three groups. However, MAO-B inhibitor use was associated with ~2-fold lower change in daily dose of levodopa (*p* < 0.001) and lower dyskinesia scores (*p* = 0.028) than non-users. No intra-class differences were observed between selegiline and rasagiline. Long-term use of MAO-B inhibitors resulted in a significant reduction in levodopa requirements and a lower frequency of dyskinesias in patients with PD. Selegiline and rasagiline had equal efficacy in controlling motor symptoms in PD patients on optimized therapy.

**Electronic supplementary material:**

The online version of this article (doi:10.1007/s00415-017-8523-y) contains supplementary material, which is available to authorized users.

## Introduction

Levodopa is the gold standard of pharmacological therapy for Parkinson’s disease (PD). However, for the last 25 years neurologists have delayed the introduction of levodopa therapy on the understanding that treatment duration was associated with an increased risk of dyskinesias. Recent evidence suggests that the risk of motor complications correlates with disease progression and daily levodopa dose, independent of treatment duration [4, 12, 13]. Therefore, modern ‘Levodopa-sparing strategies’ should focus on reducing daily levodopa dose rather than withholding its introduction. Monoamine oxidase type B (MAO-B) inhibitors are chemical agents indicated for prolonging the anti-Parkinson activity of levodopa. Symptomatic effects are due to the increase in synaptic dopamine levels and in dopamine half-life achieved by blocking its degradation. Selegiline was the first MAO-B inhibitor introduced onto the market, followed by rasagiline 15 years later [8, 13]. MAO-B inhibitors enable neurologists to delay the introduction of levodopa therapy in the early stages and to optimize the management of levodopa-related motor complications (fluctuations and dyskinesias) in more advanced stages [2, 12, 16, 23, 24].

To date, comparative analyses examining the efficacy of different MAO-B inhibitors are limited to meta-analyses and with results remaining inconsistent there is no confirmation that they improve symptoms associated with PD [17, 21, 27]. A health economics and outcomes research study comparing selegiline with rasagiline revealed that rasagiline was associated with an increase in overall cost [9]. However, in the absence of evidence supporting the superiority of rasagiline over selegiline in terms of efficacy [27], it remains challenging for clinicians to identify the most appropriate agent to prescribe.

The aim of this real-life case–control study was to compare the efficacy of selegiline with rasagiline in controlling motor symptoms over a 3-year period in patients with PD.

## Materials and methods

### Study design and patient selection

Patients included in this retrospective case–control study were selected using the Parkinson Institute (ASST G. Pini-CTO, ex-ICP, Milan) research database, which contains detailed demographic, clinical and lifestyle information on all patients assessed at the Parkinson Institute. Data from all patients seen between 1st October 2009 and 31st October 2015 and suffering from idiopathic PD diagnosed according to UK Brain Bank criteria were reviewed [15]. Patients with vascular parkinsonism were excluded on the basis of magnetic resonance brain imaging evaluation [6]. Patients treated with advanced-stage therapies, such as deep brain stimulation, continuous apomorphine infusion and levodopa duodenal infusion at baseline or during follow-up were also excluded.

**Fig. 1:**
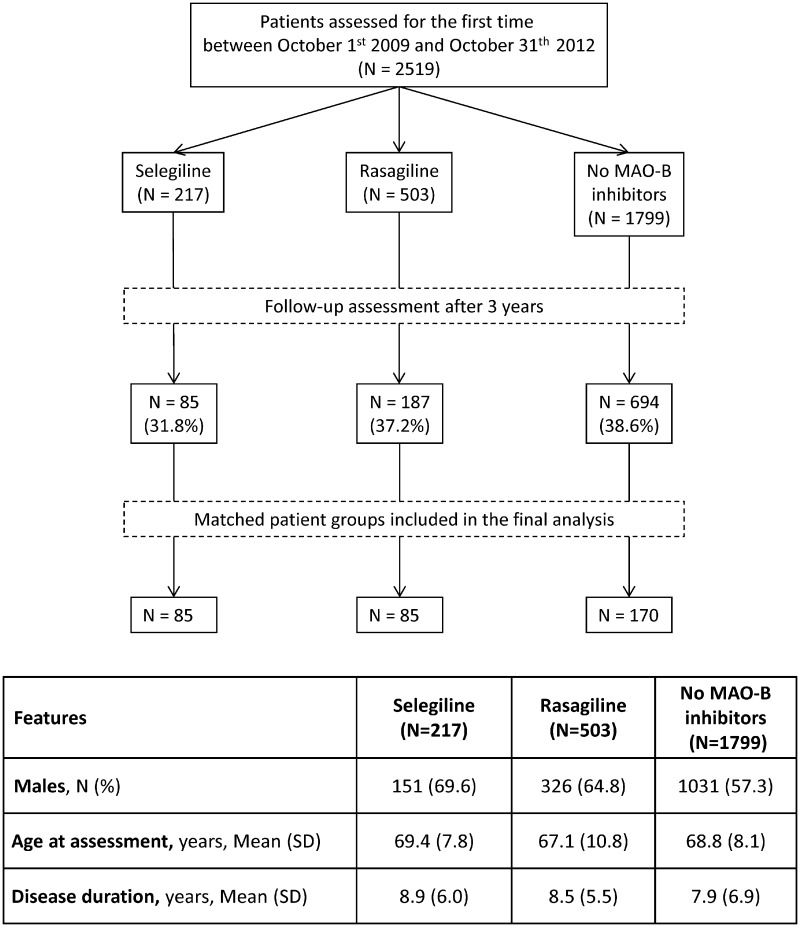
Flowchart of the study population

Information on consecutive patients who were prescribed therapy with selegiline or rasagiline between 1st October 2009 and 31st October 2012 and had a follow-up assessment at 3 years (±6 months) was extracted. Patients with PD who had been treated with selegiline (5 or 10 mg daily) for at least 3 years were selected and matched on a 1:1 ratio with patients with PD who had been treated with rasagiline (0.5 or 1 mg daily) for at least 3 years; matching was performed by gender, disease duration (±1 year) and age (±1 year) at initiation of MAO-B inhibitor therapy. Finally, the same matching procedure was applied to extract a group of patients with PD who had never received a prescription for a MAO-B inhibitor (*n* = 170). A patient flow diagram is presented in Fig. 1.

### Patient demographics and study endpoints

General demographic information (age, years of education and smoking status) and clinical data at baseline and 3 years of follow-up were extracted from the database and analyzed. In agreement with a recent clinical report, relevant clinical features were defined according to established clinical diagnostic criteria, Unified Parkinson Disease Rating Scale (UPDRS) item scores, and current pharmacological therapy (Table 1) [1, 3, 6].

**Table 1:** Definition of clinical features Adapted from Cilia et al. [6]

Clinical feature	Definition
Scoring of dopaminergic deficiency	UPDRS-Part III (in medication-on condition), sum of items 19–22, 24–26 [20]
Scoring of predominantly non-dopaminergic deficiency	UPDRS-Part III (in medication-on condition), sum of items 18, 27–30 [20]
Non-motor symptoms	
Dementia	UPDRS-Part I, item 1 score (DSM-IV-TR criteria) [3]
Psychosis	UPDRS-Part I, item 2 score (relevant if ≥2 or required the use of specific medications) Defined as hallucinations and/or delusions, but not confusion
Depression and apathy	UPDRS-Part I, sum of item 3 + item 4 score (relevant if ≥4 or required the use of specific medications)
Orthostatic hypotension	UPDRS-Part IV, items 42 Present if the patients required the use of specific medication and/or a fall in systolic blood pressure of at least 20 mmHg and diastolic blood pressure of at least 10 mmHg within 3 min of standing was recorded (present, score = 1) [1]
Non-levodopa-responsive motor symptoms	
Dysphagia	UPDRS-Part II (in medication-on condition), item 7 score (relevant if ≥2)
Frequent falls	UPDRS-Part II (in medication-on condition), item 13 score (relevant if ≥2)
Freezing of gait	UPDRS-Part II (in medication-on condition), item 14 score (relevant if ≥2)
Postural instability	UPDRS-Part III (in medication-on condition), item 30 score (relevant if ≥2)
Motor complications	
Dyskinesias score	UPDRS-Part IV, sum of items 32–35 Defined as abnormal involuntary movements, including chorea and dystonia, that could be peak dose or diphasic; off-related dystonia was not included
Presence of dyskinesias	UPDRS-Part IV, items 32 (present, score ≥1]
Disabling dyskinesias	UPDRS-Part IV, items 33 [present, score ≥2]
OFF state score	UPDRS-Part IV, sum of items 36–39
Presence of fluctuations	UPDRS-Part IV, items 39 (present, score ≥1)

*DSM-IV-TR* diagnostic and statistical manual of mental disorders, fourth edition, text revision, *UPDRS* Unified Parkinson’s Disease Rating Scale

The primary outcome was to evaluate and compare the long-term efficacy of the MAO-B inhibitors selegiline and rasagiline. Efficacy was defined as optimization of symptoms, and clinical assessment was based on the UPDRS from Part I to Part IV [11] and the Hoehn and Yahr (HY) staging system [14]. In addition, single items of UPDRS motor examination (Part III) were used to distinguish dopaminergic from non-dopaminergic deficiency and calculate a sub-score indicative of dopaminergic (facial expression, tremor, rigidity, and bradykinesia) and predominantly non-dopaminergic (speech and axial impairment) deficiency [20]. All pharmacological therapies were also reviewed to calculate the total levodopa daily dose (in mg/day and mg/kg/day) and total levodopa equivalent doses (LEDD; including equivalent doses of other anti-parkinsonian medications [28]). Accordingly, changes in levodopa-related motor complications were also considered as study endpoints.

### Statistical analysis

Analyses were performed with the software STATA 13 (StataCorp, College Station, TX, USA). Two-tailed *p* values <0.05 indicated statistical significance. To account for multiple comparisons, it was calculated that at least 85 patients in each group were required to detect a meaningful difference in the change in UPDRS scores and HY stage at 3 years. Due to the lack of preliminary data on a similar study design, this was based on a power of 80% [Type II error (*β*)], a medium effect size (standardized difference between two means) of 0.5 [7] and a two-tailed test with a 1.7% significance level [Type I error (*α*)].

Descriptive statistics of categorical variables were presented as counts and percentages, while continuous variables were reported as mean and standard deviation or median and inter-quartile range [25th–75th percentile (inter-quartile range, IQR)] according to the normality of distribution (checked using the Kolmogorov–Smirnov test). Between-group comparisons of baseline features were performed using ANOVA (normal continuous variables) or Kruskal–Wallis test (non-normal continuous variables) and conditional logistic regression (discrete variables). Mean changes from baseline of the various UPDRS scores (parts and items) were estimated using an analysis of covariance model and compared between groups using a mixed model for repeated measures. All models were adjusted for respective baseline scores and concomitant therapy (at baseline and introduced during follow-up). A secondary analysis was also conducted to evaluate the possible interaction of age (≤57 vs >57 years) and disease duration (<6 vs ≥6 years) at assessment using median values. Finally, conditional logistic regression models were built to investigate the risk of having motor fluctuations and dyskinesias at the 3-year follow-up visit. Odds ratios and 95% confidence intervals were computed accordingly.

## Results

Baseline clinical features and therapy regimen of the study groups were comparable (Table 2). About 80% (*n* = 70) of patients treated with selegiline were prescribed half the recommended (5 mg/day) dose whilst in the other MAO-B inhibitor group, all patients received the maximum and recommended dose of rasagiline (1 mg/day) [24].

**Table 2:** Patient baseline characteristics and clinical profiles

Variable	Selegiline (*n* = 85)	Rasagiline (*n* = 85)	No MAO-B inhibitor (*n* = 170)	*p* value^e^
Males, *N* (%)^a^	56 (65.9)	56 (65.9)	112 (65.9)	1.000
Current smoking, *N* (%)	9 (10.6)	10 (11.8)	19 (11.2)	0.887
Education (years), mean (SD)	11.2 (4.4)	10.7 (4.5)	10.7 (4.3)	0.660
Age at onset of disease (years), mean (SD)	56.5 (10.0)	56.3 (9.6)	56.7 (9.6)	0.952
Age at assessment (years), mean (SD)^a^	63.1 (8.8)	62.8 (8.3)	63.2 (8.3)	0.938
Disease duration (years), mean (SD)^a^	6.6 (5.4)	6.5 (5.2)	6.5 (5.3)	0.989
UPDRS score^b^				
Part I, mean (SD)	1.0 (1.3)	1.0 (1.2)	1.2 (1.2)	0.558
Part II, mean (SD)	7.4 (4.5)	7.3 (4.7)	8.1 (5.1)	0.361
Part III, mean (SD)	15.2 (8.0)	14.8 (6.7)	15.1 (9.0)	0.945
Part IV, mean (SD)	1.9 (3.2)	1.9 (2.2)	2.0 (2.5)	0.940
Total, mean (SD)	25.5 (13.0)	26.0 (12.8)	26.4 (14.7)	0.886
Dopaminergic deficiency score^c^, mean (SD)	9.2 (5.7)	8.9 (4.5)	9.4 (6.1)	0.800
Non-dopaminergic deficiency score^c^, mean (SD)	3.2 (1.9)	3.2 (2.0)	2.9 (2.4)	0.450
Hoehn–Yahr stage, mean (SD)	1.9 (0.5)	1.8 (0.4)	2.0 (0.7)	0.341
Therapy				
LEV dose				
(mg/day), mean (SD)	348 (285)	341 (287)	348 (280)	0.891
(mg/kg/day), mean (SD)	4.5 (3.7)	4.5 (4.1)	4.7 (4.1)	0.699
Concomitant DA, *n* (%)	60 (70.6)	62 (72.9)	121 (71.2)	0.947^‡^
				0.762^#^
Concomitant COMT inhibitors, *n* (%)	12 (14.1)	13 (15.3)	37 (21.8)	0.174^‡^
				0.226^#^
LEV dose adjusted for COMT inhibitors				
(mg/day), mean (SD)	365 (316)	368 (345)	375 (334)	0.971
(mg/kg/day), mean (SD)	4.7 (4.1)	4.8 (4.6)	5.1 (4.5)	0.759
LEDD from DA (mg/day), mean (SD)	125 (117)	134 (121)	110 (98)	0.221
Total LEDD (mg/day), mean (SD)	487 (282)	513 (335)	501 (352)	0.492
Non-motor symptoms				
Dementia, *n* (%)	2 (2.4)	3 (3.7)	4 (2.4)	0.947^‡^
				0.587^#^
UPDRS-cognition item, mean (SD)	0.20 (0.40)	0.18 (0.42)	0.24 (0.43)	0.523
Psychosis^d^, *n* (%)	2 (2.4)	4 (4.9)	7 (4.2)	0.841^‡^
				0.822^#^
UPDRS-psychosis, mean (SD)	0.15 (0.39)	0.13 (0.41)	0.20 (0.42)	0.384
Depression and apathy^d^, *n* (%)	3 (3.7)	4 (4.9)	8 (4.9)	0.382^‡^
				0.995^#^
UPDRS-depression and apathy, mean (SD)	0.66 (1.02)	0.67 (1.01)	0.73 (1.02)	0.839
Orthostatic hypotension^d^, *n* (%)	2 (2.4)	4 (4.9)	11 (6.7)	0.173^‡^
				0.572^#^
UPDRS-orthostatic hypotension, mean (SD)	0.03 (0.16)	0.06 (0.24)	0.09 (0.28)	0.175
Non-levodopa-responsive symptoms				
Dysphagia, *n* (%)	1 (1.2)	2 (2.4)	3 (2.4)	0.699^‡^
				0.755^#^
UPDRS-dysphagia, mean (SD)	0.07 (0.26)	0.07 (0.35)	0.14 (0.39)	0.187
Frequent falls, *n* (%)	0 (0.0)	0 (0.0)	4 (4.7)	0.998^‡^
				0.998^#^
UPDRS-frequent falls, mean (SD)	0.08 (0.24)	0.07 (0.42)	0.15 (0.65)	0.108
Freezing of gait, *n* (%)	4 (4.7)	3 (3.5)	12 (7.1)	0.498^‡^
				0.271^#^
UPDRS-freezing of gait, mean (SD)	0.26 (0.54)	0.14 (0.42)	0.27 (0.65)	0.141
Postural instability, *n* (%)	4 (4.7)	2 (2.4)	7 (4.1)	0.823^‡^
				0.478^#^
UPDRS-postural instability, mean (SD)	0.44 (0.63)	0.49 (0.53)	0.34 (0.56)	0.113
Motor complications				
Dyskinesias score, mean (SD)	0.4 (1.0)	0.4 (1.0)	0.6 (1.2)	0.250
Dyskinesias, *n* (%)	18 (21.2)	17 (20.0)	43 (25.3)	0.523^‡^
				0.357^#^
OFF state, mean (SD)	0.6 (1.0)	0.7 (1.1)	0.9 (1.2)	0.107
Fluctuations, *n* (%)	27 (31.8)	28 (32.9)	64 (37.6)	0.404^‡^
				0.473^#^

*COMT* catechol-*O*-methyltransferase, *DA* dopamine agonists, *MAO-B inhibitors* monoamine oxidase type B inhibitors, *LEDD* levodopa equivalent daily dose, *LEV* levodopa, *SD* standard deviation, *UPDRS* Unified Parkinson’s Disease Rating Scale

^a^Matching variable

^b^In medication-on condition

^c^Calculated from UPDRS motor examination (Part III) as proposed by Levy et al. [20]

^d^Including both treated and untreated cases

^e^According to analysis of variance (continuous variables) or conditional logistic regression (discrete variables; ^‡^ selegiline vs. no MAO-B, ^#^ rasagiline vs. no MAO-B) as appropriate

**Fig. 2:**
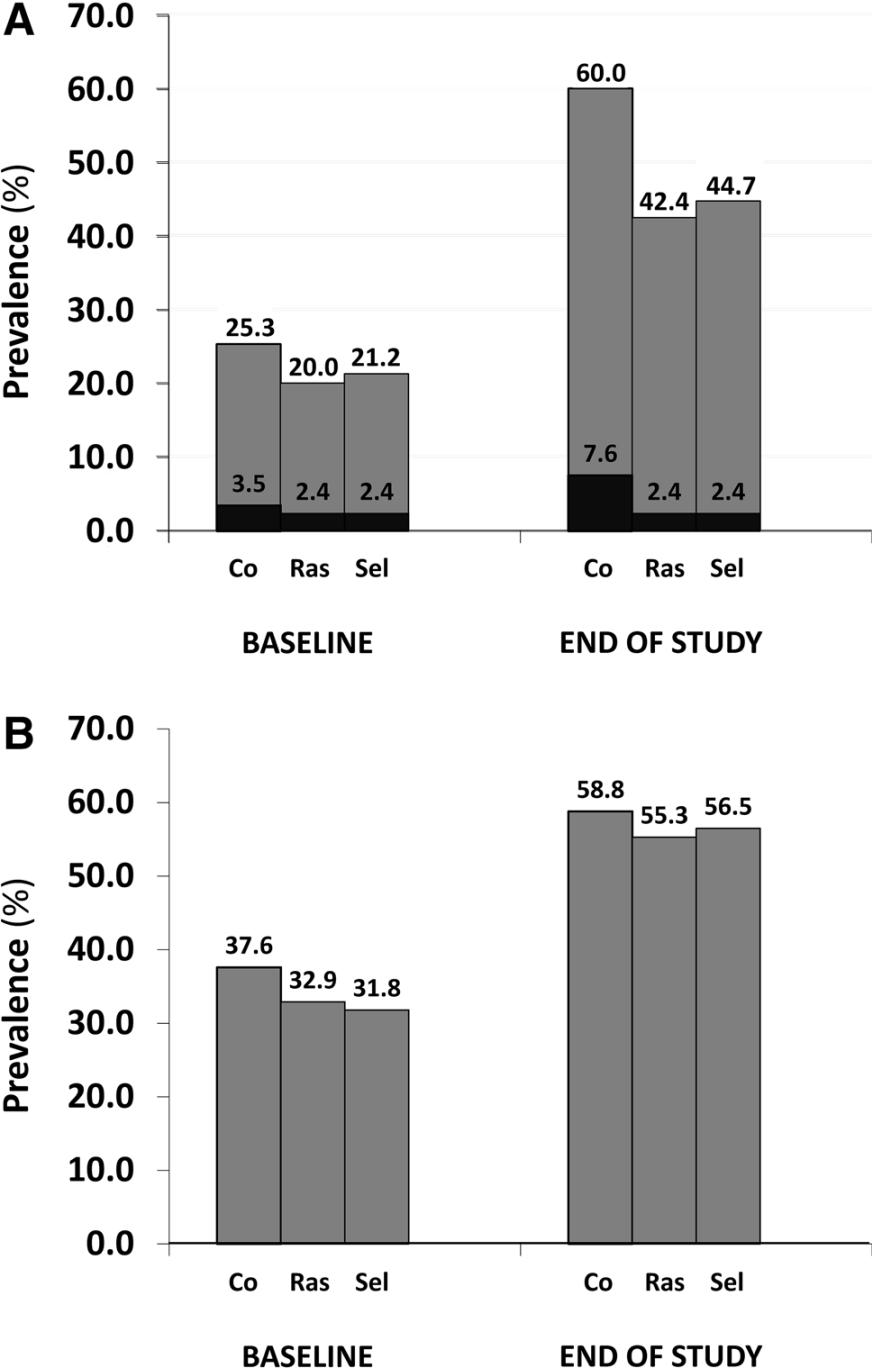
Prevalence of dyskinesias [**a** disabling dyskinesias (UPDRS Part IV-items 33 score ≥2) are highlighted in *black color*] and motor fluctuations (**b**) in the study population. Controls (Co.) indicate patients who did not receive any monoamine oxidase type B (MAO-B) inhibitor (*Ras* rasagiline, *Sel* selegiline)

After a mean follow-up of about 37–38 months, there was no difference in the hallmarks of clinical progression of PD between MAO-B inhibitor users and non-users or between rasagiline and selegiline users (Table 3). This applied to both motor and non-motor symptoms, and motor symptoms reflecting predominant dopaminergic and non-dopaminergic deficiency. Interaction analysis showed that this lack of effect was not modified by age or disease duration at the time of introduction of MAO-B inhibitor therapy. However, the use of MAO-B inhibitors was associated with a lower increase in the UPDRS scores for dyskinesias (*p* = 0.028; Table 3) and lower prevalence of dyskinesias at follow-up (Fig. 2, plot a), with an OR for rasagiline of 0.47 (95% CI 0.28–0.81; *p* = 0.006) and 0.53 for selegiline (95% CI 0.31–0.90; *p* = 0.019). The same applied to incident dyskinesias during follow-up: for rasagiline, 0.54 (95% CI 0.29–0.98; *p* = 0.045); for selegiline, 0.56 (95% CI 0.30–0.99; *p* = 0.049). A trend to significance was found for prevalent troublesome dyskinesias at the end of study [for rasagiline, 0.29 (95% CI 0.06–1.33), *p* = 0.111; for selegiline, 0.30 (95% CI 0.07–1.35), *p* = 0.116]. No difference in prevalent motor fluctuations at follow-up was observed (Fig. 2, plot b), with an OR for rasagiline of 0.82 (95% CI 0.48–1.39; *p* = 0.457) and 0.84 for selegiline (95% CI 0.49–1.44; *p* = 0.529). The use of MAO-B inhibitors for 3 years was associated with a significant reduction in levodopa daily dose (*p* < 0.001; Supplementary Table 1), with non-users requiring about a twofold higher increase in dose (expressed in mg/kg/day) of either levodopa alone or levodopa adjusted for the use of catechol-*O*-methyltransferase inhibitors (*p* < 0.001 for both) at follow-up. There was no intra-class difference in terms of levodopa dose reduction. Overall, there was no difference in the total amount of LEDD either between MAO-B inhibitors users and non-users, or between the two MAO-B inhibitor drugs.

**Table 3:** Follow-up clinical data of the study population by use of monoamine oxidase type B inhibitors

Variable	Selegiline (*n* = 85)	Rasagiline (*n* = 85)	No MAO-B inhibitor (*n* = 170)	*p* value^c^
Follow-up duration (months), mean (SD)	37.5 (7.6)	37.9 (7.5)	37.6 (6.8)	0.931
Change in UPDRS score^a^				
Part I, mean (SD)	0.18 (0.15)	0.15 (0.16)	0.21 (0.12)	0.718
Part II, mean (SD)	1.99 (0.33)	2.54 (0.34)	1.97 (0.25)	0.368
Part III, mean (SD)	3.72 (0.58)	4.54 (0.58)	4.07 (0.41)	0.598
Part IV, mean (SD)	0.88 (0.23)	0.83 (0.24)	1.27 (0.18)	0.092
Total, mean (SD)	6.77 (0.91)	8.06 (0.90)	7.52 (0.57)	0.333
Dopaminergic deficiency score^b^, mean (SD)	2.16 (0.42)	2.83 (0.42)	2.32 (0.30)	0.482
Non-dopaminergic deficiency score^b^, mean (SD)	1.15 (0.22)	1.30 (0.22)	1.29 (0.16)	0.857
Hoehn–Yahr stage, mean (SD)	0.26 (0.05)	0.37 (0.05)	0.30 (0.03)	0.304
Increase in stage, *n* (%)	33 (38.8)	40 (47.1)	64 (37.6)	0.279
Change in therapy				
LEV dose				
(mg/day), mean (SD)	136 (23)	107 (22)	217 (16)*	<0.001
(mg/kg/day), mean (SD)	1.74 (0.30)	1.43 (0.29)	2.93 (0.21)*	<0.001
New association of DA, *n* (%)	3 (3.5)	6 (7.0)	11 (6.5)	0.313^‡^
				0.867^#^
New association of COMT inhibitors, *n* (%)	8 (9.4)	8 (9.4)	19 (11.2)	0.645^‡^
				0.661^#^
LEV dose adjusted for COMT inhibitors				
(mg/day), mean (SD)	153 (29)	116 (26)	239 (18)*	**<0.001**
(mg/kg/day), mean (SD)	1.96 (0.37)	1.59 (0.36)	3.23 (0.26)*	**<0.001**
LEDD from DA (mg/day), mean (SD)	11.7 (9.3)	20.9 (9.2)	19.6 (6.4)	0.601
Total LEDD (mg/day), mean (SD)	249 (29)	260 (28)	271 (20)	0.502
Change in non-motor symptoms				
UPDRS-cognition item, mean (SD)	0.06 (0.05)	0.05 (0.05)	0.10 (0.04)	0.252
UPDRS-psychosis, mean (SD)	0.04 (0.06)	0.05 (0.06)	0.09 (0.04)	0.744
UPDRS-depression and apathy, mean (SD)	0.05 (0.11)	0.05 (0.12)	0.09 (0.09)	0.942
UPDRS-orthostatic hypotension, mean (SD)	0.01 (0.03)	0.06 (0.03)	0.01 (0.03)	0.387
Change in non-levodopa-responsive symptoms				
UPDRS-dysphagia, mean (SD)	0.03 (0.03)	0.06 (0.04)	0.08 (0.04)	0.300
UPDRS-frequent falls, mean (SD)	0.10 (0.05)	0.05 (0.05)	0.02 (0.04)	0.159
UPDRS-freezing of gait, mean (SD)	0.22 (0.06)	0.15 (0.06)	0.27 (0.05)	0.316
UPDRS-postural instability, mean (SD)	0.26 (0.08)	0.30 (0.06)	0.43 (0.30)	0.138
Change in motor complications				
Dyskinesias score, mean (SD)	0.27 (0.11)	0.22 (0.11)	0.52 (0.07)*	**0.028**
OFF state, mean (SD)	0.58 (0.10)	0.53 (0.10)	0.61 (0.07)	0.407

*COMT* catechol-*O*-methyltransferase, *DA* dopamine agonists, *iMAO-B* monoamine oxidase type B inhibitors, *LEDD* levodopa equivalent daily dose, *LEV* levodopa, *SD* standard deviation, *UPDRS* Unified Parkinson’s Disease Rating Scale

^a^In medication-on condition

^b^Calculated from UPDRS motor examination (part III) as proposed by Levy et al. [20]

^c^According to analysis of variance (continuous variables; * significantly different from the other groups by pairwise comparison) or conditional logistic regression (discrete variables; ^‡^ selegiline vs. no MAO-B, ^#^ rasagiline vs. no MAO-B) as appropriate

## Discussion

This is the first head-to-head real-life study to compare the efficacy of selegiline and rasagiline in terms of clinical features, motor complications and therapy regimen. Data indicate that long-term use of MAO-B inhibitors during the mid-stages of PD results in a significant reduction in levodopa requirements and a lower frequency of dyskinesias. The results do not illustrate a significant advantage of MAO-B inhibitors in the control of motor symptoms in patients on optimized medical therapy. After 3 years, UPDRS Part I, II and III scores in patients treated with selegiline were similar to those in patients treated with rasagiline, reflecting equal efficacy of the two medications on non-motor symptoms, activities of daily living, and motor symptoms. Moreover, treatment with MAO-B inhibitors was associated with a levodopa dose reduction of about 70–100 mg/day compared with patients who had never been treated with any MAO-B inhibitor, independently of the compound used. Incidence of dyskinesias was also significantly lower amongst MAO-B inhibitor users than non-users at the end of follow-up. These data suggest that combination therapy is an effective ‘levodopa-sparing strategy’ and pharmacological therapy may be optimized to achieve a better control of disability associated with motor and non-motor symptoms, ultimately reducing the risk of dyskinesias.

According to a recent meta-analysis, the use of MAO-B inhibitors as adjunct therapy to levodopa in the early stages of PD enables a reduction in levodopa dose of about 30 mg/day, but no difference in the frequency of dyskinesias was recorded compared with placebo [27]. In the PD-MED trial [13], no difference was found in the rate of dyskinesias. The DATATOP study reported a higher rate of dyskinesias in patients receiving selegiline, at least for those who had experienced these events during the initial part of the trial [26]. The data presented in this report may appear to contradict these previous findings, given that a significantly lower rate of dyskinesias was observed in patients treated with MAO-B inhibitors. A number of factors could account for this discrepancy. The PD-MED trial compared different first-line treatment strategies in newly diagnosed patients, and those allocated to MAO-B inhibitors or dopamine agonists were prescribed additional levodopa to optimize symptoms as necessary. Interestingly, patients receiving MAO-B inhibitors were more likely than those allocated levodopa to need a drug from another class added to their treatment. No specific directions were provided on the amount of levodopa to be used. As a consequence, total daily LEDD at 7 years in patients allocated to receive MAO-B inhibitors was 60 mg higher than in those randomized to levodopa. In the present study, the 3-year observation period was initiated after a mean of 6.5 years after the onset of PD, when motor fluctuations and dyskinesias are more likely. Indeed, it has been previously reported in a population of patients with PD with limited access to medication that mean disease duration at wearing-off was approximately 5.5 years, while it was 6.5 years at the onset of dyskinesias [4]. At baseline, approximately 32% of patients already had motor fluctuations and 18% already had dyskinesias, which indicates that new (incident) cases of patients experiencing motor complications (wearing-off phenomenon and dyskinesias) over a 3-year period were detected with more sensitivity than in trials involving patients with early untreated PD. MAO-B inhibitors can be used both as monotherapy in the early stages of the disease and as adjuvant therapy to levodopa in more advanced stages, and the present study has the advantage of focusing broadly on the use of these drugs in daily clinical practice. Interestingly, total LEDD at 7 years in the PD-MED trial was also significantly higher than in the present study’s population at baseline (~700 vs. ~500 mg/day). Although a difference in mean body weight between the UK and Italy does exist (higher in the UK) [10], and selection bias cannot be fully excluded, it is possible to speculate that in the advanced stages of the disease, better optimization of the daily levodopa dose could be achieved in association with MAO-B inhibitors. The present findings may be also the result of a rigorous optimization of therapy at the Parkinson Institute, with the prescription of available pharmaceutical agents at the lowest dose required to achieve clinical benefit, as endorsed by the recent literature [4, 12]. Interestingly, use of drugs from other classes of anti-Parkinson agents was similar across groups. This report further supports the use of levodopa–MAO-B inhibitor combination therapy in patients with PD as it allows a reduction of levodopa daily dose and, consequently, the overall risk of dyskinesias in the long term [4, 13].

Previous meta-analyses on intra-class efficacy in the treatment of early PD yielded conflicting results [17, 21, 27]. Although the meta-analysis based on the most selective literature search revealed that both drugs were equally effective in controlling motor symptoms [21], another suggested a possible difference between the two MAO-B inhibitors in favor of selegiline, in terms of reduction in daily levodopa dose [27]. In this real-life 3-year case–control study we found no intra-class difference.

Preclinical evidence has supported clinical studies in the extensive search for possible disease-modifying effects and neuroprotective properties of MAO-B inhibitors. This hypothesis was supported by in vitro and in vivo preclinical studies [24], as well as a large clinical trial, the DATATOP trial, which showed a sustained effect of selegiline in delaying the onset of disability requiring levodopa treatment [2, 25]. Although the interest in the potential disease-modifying effects of MAO-B inhibitors has increased, and the effects of rasagiline in patients with early PD have been investigated [22], their use in this indication has not been approved by the Food and Drug Administration (FDA). Several large clinical trials investigated rasagiline [2, 23, 24]. However, the interpretation of the results from these studies is complex and confounded by the symptomatic effect of these drugs and the inadequacy of the follow-up schedule, which was too short to provide meaningful results [24]. In the present study, we found no differences between MAO-B inhibitor users and non-users in the total UPDRS score and the severity of all motor and non-motor symptoms investigated at the end of follow-up, including major milestones of disease progression [19]. Furthermore, the assessment of disease progression represented by total UPDRS score is in line with previous randomized clinical trials [2, 22, 23]. In particular, the large ADAGIO study showed a 3-point change in the UPDRS score over an 18-month period [22], while a 7-point change over a 36-month period is reported here; it is worth noting that previous large trials have included patients in the early stages of PD [2, 23]. However, the results of the present real-life study do not warrant a conclusive statement about the potential disease-modifying effects of MAO-B inhibitors. This is due to one of the limitations of our study, namely that patients were in the medication-ON state as opposed to the medication-OFF state, in which disability and disease severity should be assessed. However, from an ethical stance an adequate wash-out was not possible considering that the irreversible enzyme inhibition from both MAO-B inhibitors requires at least a 3-week wash-out. Furthermore, evaluation in the “practical OFF” state would have been an additional source of bias in the interpretation of motor performance, as true wash-out from drugs after 12 h could not be obtained for dopamine agonists (which are all prolonged-release formulations, 5 half-lives are required for wash-out) and levodopa, even in the advanced stages of the disease [5, 29]. Another limitation of this study is the fact that, because it is a retrospective analysis of longitudinal data, prescription bias cannot be fully excluded. Clinicians may prefer to prescribe one MAO-B inhibitor over another, and this choice is likely to have a subjective component, as well as depend on demographic, clinical, and/or therapeutic characteristics of patients with PD [9]. However, baseline clinical characteristics of treatment groups were comparable in this study. Although different from that administered in previous studies, the dose of daily selegiline used in most patients (5 mg) was determined to achieve clinical benefit whilst minimizing risk. This further enhances the value of the present findings. Nonetheless, the results of the PD-MED trial have shown that in a real-life setting the dose of selegiline is frequently below recommended (mean dose during follow-up: 8.5 mg/day) [13]. Finally, sample size may appear to confer limited power to the present study compared with previous trials; however, based on results from meta-analyses, thousands of patients would be required for a randomized-controlled trial to detect a few-point difference in disease severity rating scales, the clinical significance of which remains questionable.

Strengths of this study were the stringent matching criteria and the clinical setting. The Parkinson Institute is a large tertiary-care center where patients are assessed over time by the same neurologist experienced in movement disorders, which is reflected by a comprehensive clinical assessment of motor and non-motor symptoms and the prescription of optimized therapeutic strategies. In the absence of direct comparisons of MAO-B inhibitors in phase-III trials, phase-IV studies are valuable. The observation period (37–38 months) was relatively long compared with the DATATOP (up to 24 months; mean follow-up 14 months) [2] and the ADAGIO (18 months) [22] trials, but shorter than the PD-MED (up to 7 years; median 3 years) [13] and PDRG-UK (up to 14 years) [18] trials, though the latter two did not examine the efficacy of different MAO-B inhibitors.

## Conclusions

Long-term use of MAO-B inhibitors in patients in the mid-stages of PD led to a significant reduction in levodopa requirements and lower frequency of dyskinesias, independently of which MAO-B inhibitor was administered, selegiline or rasagiline. The results of this study suggest that the MAO-B inhibitors selegiline and rasagiline have similar efficacy in controlling motor symptoms in patients with PD on optimized therapy.

## Electronic supplementary material

Below is the link to the electronic supplementary material.

Supplementary material 1 (DOC 41 kb)
