# Supplementary material for: Efficacy of rasagiline and selegiline in Parkinson’s disease: a head-to-head 3-year retrospective case–control study
Source: J Neurol. 2017 May 26;264(6):1254–63. doi: 10.1007/s00415-017-8523-y (PMC5570795; doi:10.1007/s00415-017-8523-y)
Supplement: Supplementary file 1 — Supplementary material 1 (DOC 41 kb) [file 415_2017_8523_MOESM1_ESM.doc]

# Efficacy of rasagiline and selegiline in Parkinson’s disease: a head-to-head 3-year retrospective case-control study

Emanuele Cereda MD, PhD*^1^, Roberto Cilia MD^2^, Margherita Canesi MD^2^, Silvana Tesei MD^2^, Claudio Bruno Mariani MD^2^, Anna Lena Zecchinelli MD^2^, Gianni Pezzoli MD^2^.

^1^ Nutrition and Dietetics Service, Fondazione IRCCS Policlinico San Matteo, Pavia.

^2^ Parkinson Institute, ASST G. Pini-CTO, ex-ICP, Milan, Italy.

Corresponding author: Emanuele Cereda MD, PhD; Nutrition and Dietetics Service, Fondazione IRCCS Policlinico San Matteo, Viale Golgi 19, 27100 Pavia, Italy; Tel: +39 0382 501615, Fax: + 39 0382 502801, E-mail: [e.cereda@smatteo.pv.it](mailto:e.cereda@smatteo.pv.it).

**Supplementary Table 1.** Pharmacological treatment at the end of follow-up data of the study population by use of Monoamine Oxidase type B Inhibitors

| **Variable** | **Selegiline**  (n=85)  5 mg (n=70)  10 mg (n=15) | **Rasagiline**  (n=85)  1 mg (n=85) | **No MAO-B inhibitor**  (n=170) | ***p*-value^d^** |
| --- | --- | --- | --- | --- |
| *LEV dose*  (mg/day), mean (SD) | 487 (250) | 456 (237) | 562 (290) * | **0.006** |
| *LEV dose*  (mg/kg/day), mean (SD) | 6.3 (3.2) | 6.1 (3.3) | 7.6 (4.1) * | **0.002** |
| *Concomitant DA*, n (%) | 63 (74.1) | 68 (80.0) | 132 (77.6) | 0.652 |
| *Concomitant COMT inhibitors*, n (%) | 20 (23.5) | 21 (24.7) | 56 (32.9) | 0.123 |
| *LEV dose adjusted for COMT inhibitors*  (mg/day), mean (SD) | 538 (273) | 495 (276)† | 644 (383) | **0.002** |
| (mg/kg/day), mean (SD) | 6.9 (3.8) | 6.6 (3.9) | 8.7 (5.1) * | **<0.001** |
| *LEDD from DA* (mg/day), mean (SD) | 138 (99) | 167 (142) | 132 (109) | 0.071 |
| *Total-LEDD* *excluding MAO-B inhibitor*  (mg/day), mean (SD) | 682 (286) | 675 (295) | 776 (290) * | **0.009** |
| *Total-LEDD* *including MAO- B inhibitor*  (mg/day), mean (SD) | 741 (283) | 775 (296) | 776 (290) | 0.651 |
| *LEDD from MAO- B inhibitor* (mg/day), mean (SD) | 59 (19) | 100 (0) | - | - |
| *Abbreviations*: COMT, catechol-O-methyltransferase; DA, dopamine agonists; iMAO-B, Monoamine Oxidase type B Inhibitors; LEDD, levodopa equivalent daily dose ; LEV, Levodopa; SD, standard deviation; UPDRS, Unified Parkinson’s Disease Rating Scale.  ^a^According to one-way analysis of variance (post-hoc comparison of means: * significantly different from the other groups; † significantly different from control group) | | | | |
